# Pain-associated biomarkers in breast cancer

**Published:** 2015

**Authors:** C Diaconu, C Pantis, C Cirimbei, C Bordea, MI Gruia, A Blidaru

**Affiliations:** * Anaesthesia and Intensive Care Unit, Oncological Institute Bucharest, Romania; ** 1st Department of General Surgery, “Carol Davila” University of Medicine and Pharmacy, Oncological Institute Bucharest, Romania; *** 2nd Department of General Surgery, “Carol Davila” University of Medicine and Pharmacy, Oncological Institute Bucharest, Romania; **** Biochemistry and Radiobiology Department, “Carol Davila” University of Medicine and Pharmacy, Bucharest, Romania

**Keywords:** breast cancer, pain cancer, lipid peroxides, ceruloplasmin, immune circulating complexes

## Abstract

Breast cancer represents a major public health problem, being the highest incidence neoplasia in females in Romania. The most important step in the treatment of this neoplasia is the surgical procedure; the biggest problem associated with this form of treatment in these patients is pain-related. Pain is a complex symptom with an impact on quality of life and psychology of cancer patient and can only be monitored verbally and subjectively. Consequently, the purpose of our work is to identify some biochemical parameters involved in the events cascade associated with inflammation and pain in breast cancer female patients, monitored in dynamics of anesthesia and surgical procedure. Measurements of lipid peroxides, ceruloplasmin and immune circulating complexes in mentioned dynamics have been performed. The recorded values are in concordance with the inflammatory processes and pain intensity, thus we can allege that these measurements can complete the pain-associated clinical picture in female breast cancer patients.

## Introduction

Breast cancer represents a major public health problem, being the highest incidence neoplasia in female sex in Romania [**1**]. A major factor in order to obtain favorable results in breast cancer is represented by an early diagnosis and a proper therapeutic management. The treatment decision is based on the following: stage, axillary nodes status, tumor biology, histopathological and immunohistochemical characteristics and includes the surgical procedure as an important stage [**2**]. This is associated with pain specific episodes.

Pain is frequent in cancer patients and remains one of their most important suffering aspects, besides the multiple analgesic therapy methods. Pain is a complex symptom with an impact on quality of life and psychology of the cancer patient [**3**]. Definitions of pain try to cover the big diversity of aspects of this symptom, from the simple explanation as pure physiological phenomenon, until the usual multidimensional type image. Pain is an individual experience that includes physical and psychosocial aspects and is always subjective. There are many classification modalities, that reflect the multidimensional aspect of pain sensation, but until now, there has been no universal description system [**4**]. These classifications have been elaborated in relation to the following:

- neuropsychological mechanisms

- localization of major pain sources

- temporal pain aspects

- nature of pain

- modality of pain association and response to treatment

- specific cancer pain syndromes

Related to the clinical description, pain can be:

- nociceptive: well-localized, deaf, piercing (e.g. bone, soft tissues)

- visceral: profound, diffuse, severe, possibly transmitted through profound organs implications: spastic, colicative.

- neuropathic: sudden, flashing, like an electrical shot, associated with paresthesia or dysesthesia (like a burn)

- psychosomatic: the pain of whole body, variable localization, without information about the precise location, tormenting, destructive

- sympathetic: unsegmentary, associated with thermal sensations, dysesthesia, like a burn, movement independent, associated with trophic perturbations.

Clinical expression of pain can be influenced by multiple factors, for this reason, pain treatment needs a complex approach of the patient like a person. Pain treatment is a dynamic process, which needs frequent reevaluations in order to verify the efficiency of the therapy and to facilitate the doses adjustment [**5**]. The progress of cancer disease often reclaims an increase of the analgesic doses and the opioid tolerance expresses itself like a decrease in the duration or disappearance of analgesia. An adequate treatment of secondary opioid effects is of capital importance because these represent a barrier in front of an effective analgesia obtaining. For a proper approach of this pain treatment and to objectively evaluate the intensity of its physical manifestations, in this paper we proposed to correlate pain intensity with some biochemical parameters involved in female breast cancer patients, monitored during the dynamic of anesthesia and surgical procedure.

## Materials and methods

**Patients sets**

30 female patients who gave their consent in order to participate in supplementary biochemical investigations performed in their area were enrolled. The experimental model has been exposed for approval to the ethics committee of the institute. The patients were between 45 and 65 years old. They were diagnosed with breast cancer stage II, intraductal carcinomas, untreated with chemicals or radiation before the surgical procedure. The surgical act was a radical intervention with a medium duration of 2 hours. The pain scale was verbally registered at 24 hours after the intervention, reaching a medium value of 8.9. For the pain therapy, opioids were administered in intravenous perfusion until 24 hours post-operatory, than only minor analgesics. In the anesthetic-surgical act dynamic, blood samples were harvested, by venous puncture before anesthesia, at 24 hours after the surgical procedure, at 48 hours and at 7 days after the surgical act. From the samples harvested in this dynamic, the following biochemical parameters were determined: lipid peroxides, serum ceruloplasmin and immune circulating complexes.

**Lipid peroxides determination**

Although lipid peroxidation of biological samples can be evaluated by different physical and chemical methods, based on MDA measurement (final product of lipid peroxidation reaction) formed by endoperoxides cracking during the last step of polyunsaturated fatty acids oxidation, being the simplest and largest scale utilized in present [**6**,**7**]. MDA from the serum sample is a weak, unstable, acid, very reactive, that reacts with TBA (thiobarbituric acid) on boiling, in tricloractic acid environment. The red product resulted has a maximum of absorption at 532 nm. The absorption of colored product by the precipitate is prevented by adding hydrochloric acid, and the turbidity produced by lecithin is removed with organic solvents (CHCl3 or n-butanol).

Although controversial, the method has a large utilization. The most difficult problem is the identification of carbonyl products that react with TBA, of what MDA is only a part. Studying specific extinctions at 532 nm of carbonyl compounds resulted by oxidative degradation of lipid hydroperoxides, it can be considered without error that the 532 nm absorption of TBA-MDA complex. Normal values: 0-4 mmol/ dl serum.

**Ceruloplasmin determination**

The physiological role of ceruloplasmin is complex and intensively researched. Among these, there are:

- copper ions transportation

- organic amines oxidation

- oxidation of Fe 2+ to Fe 3+ after releasing it from transferrin and ferritin

- antioxidative activity related to lipid peroxidation

-endogenous modulation of inflammatory response

- stimulation of cellular proliferation and angiogenesis.

Ceruloplasmin (CP) is an acute phase protein with an intermediary response in comparison with other acute phase proteins, whose level increases 2-3 times in inflammations, pregnancy, traumatic lesions and surgical procedures. Serum ceruloplasmin level is useful as a clinical indicator in Wilson disease. Every CP molecule contains 6 or 7 copper atoms and 90-95% of serum copper can be found in this form [**8**].

Studies on humans and animals showed that copper deficiency increases the plasma cholesterol and LDL-cholesterol level, but decreases HDL-cholesterol, which increases the cardiovascular disease risk.

The liver is the major site for ceruloplasmin synthesis, but it can also be extrahepatically synthesized especially in the lungs.

Ceruloplasmin is a light-blue-colored protein with oxidative activity on the polyamines, polyphenols and inorganic ferrous ions (Fe 2+). The main biological substratum is Fe 2+, ceruloplasmin having a maximum affinity for this. Catalytic oxidation of Fe 2+ or complexes containing Fe 2+ is named ferroxidase activity. Several methods for the determination of ceruloplasmin ferroxidase activity are described. Ravin method that consists in the reaction of p-phenylenediamine in acetic acid - acetate buffer was used [**9**].

The enzyme quantity that converts 1 µmol substratum per minute is defined as 1 unit of ceruloplasmin.

Normal values: 80-120 U.I. ceruloplasmin.

Auto oxidation of Fe 2+ is inhibited during testing and keeping samples, using thiourea, without ferroxidase activity.

**Immune circulating complexes determination**

Immune circulating complexes are protein aggregates formed by the binding of antigens with antibodies. They are continuously forming as a result of the immune response. Because they are fast destroyed in the blood circulation, these immune complexes (CIC) are not detected in serum or they are detected in very small quantities. CIC detection is possible in some conditions in which there exists a high immune complex concentration that exceeds phagocytes clearance [**10**]. CIC increasing conducts to an increase of thrombocytes and erythrocytes aggregation. In addition, because of complement system activation, inflammatory reactions may appear, that determine leucocytes stimulation, macrophages activation with consecutive alteration at tissular level. This complement activation can elicit a series of potentially destructive events, like cell lysis, anaphylatoxins production, leucocitaria stimulation and macrophages activation. The majority of the tissular lesions are induced by immune complexes fixation at cell membranes level [**11**]. Although CIC detection is not specific for one condition, CIC testing can provide interest information about the pathology, disease course and prognosis. CIC presence is an indicator about the exceeded immune defense or autoimmune conflict that conducts to the necessity of performing some complementary tests to clarify the diagnosis. Highly persistent concentrations of CIC may indicate an active chronic disease, while CIC concentration normalization indicates the treatment success [**12**].

We determined CIC by modified Haskova method. Normal values are between 1-50 µmoli/ ml. The test is useful for monitoring the diseases with immune complexes (SLE, glomerulonephritis, vasculitis, serum disease), as well as monitoring some neoplasias evolution [**13**].

## Results and discussion

The results are presented in the following figures. We registered an increase of the lipid peroxides level with a maximum peak at 48 hours after the surgical procedure, the curve profile showing an increase of values until 7 days post-operatory. These data are in accordance with the VAS pain scale. From a biochemical point of view, data confirm mechanisms that are based on malignization and pain occurrence. In case of cell modification induced by malignization, eicosanoides, especially those PGE2-type are induced by the angiogenesis basic regulators, β-TGF, endothelin-1 [**14**]. Recent studies have shown that COX-2 and oncoprotein p53 are fundamental markers of angiogenic process. Associated with p21ras oncogene, the promoting effect of polyunsaturated n-6 fatty acids in experimental carcinogenesis of mammary gland can be explained [**15**]. These polyunsaturated fatty acids are spread in cell membranes, where they are exposed to lipid peroxidation reaction. The most frequent acid is the arachidonic acid. This acid is also present in membranes as a compound of phosphatidylinositol, phosphatidylserine, triacylglycerols (from adipose tissue) and cholesterol esters (localized especially in suprarenal and ovaries). Therefore, the first step of biosynthesis consists of releasing the arachidonic acid from cell membranes that take place under the action of phospholipase A2. This enzyme, compound of cell membranes, must be activated under the action of some immunological stimuli like phagocytosis, antibodies, immune complexes, bacterial endotoxins, and limphokines [**16**]. As phospholipases A2 type I is tightly related to the membranes, being stimulated by Ca2+, at physiological pH, that of type II, cytoplasmatic, is inhibited by the calcium ions and it has an optimal pH at acid values. Arachidonic acid can also be released by the thrombin from thrombocytes and by the contracting of the aortic factor in the lungs, etc. The most interesting are the apparently contradictory actions that can be understood and explained in the context of inflammatory process. Cox-2 is the prevailing isoform of cyclooxygenases, present at the inflammation spot and which produce prostaglandins that cause swelling and pain. In situations when the release of protective prostaglandins by Cox-1 was lost, Cox-2 induction can compensate and reduce the inflammatory response. Therefore, Cox-2 is anti-inflammatory in the case of cell proliferation [**17**]. Cox-2 is related to inflammatory cell and tissue types and is believed to be the target enzyme for nonsteroidal drugs anti-inflammatory activity. This way, the relation between inflammation and lipid peroxidation is unquestionable and we succeeded to prove, by these determinations, the pain intensity produced by inflammation and lipid peroxidation in investigated patients.

**Fig. 1:**
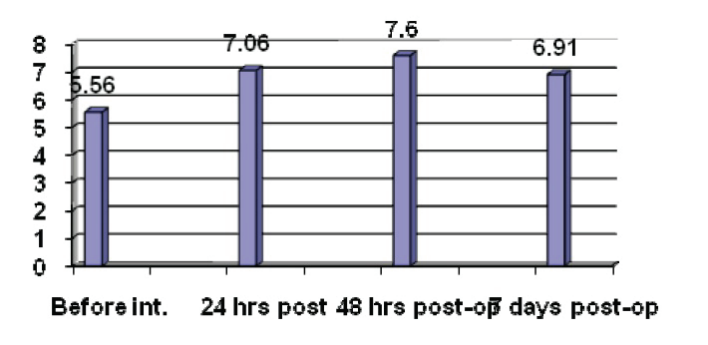
Dynamic of lipid peroxides values during anesthesia and surgical procedure

**Fig. 2:**
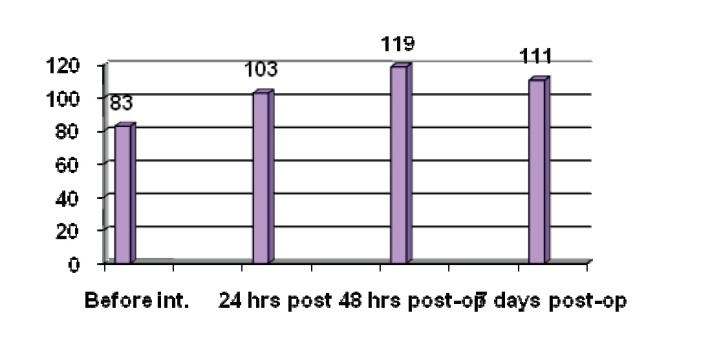
Dynamic of ceruloplasmin values during anesthesia and surgical procedure

Copper ions are powerful catalysers of free radicals lesions. They convert H2O2 to OH-, decomposing lipid peroxides; catalyze auto oxidative reactions, especially of ascorbate, DNA oxidation and LDL peroxidation. The median copper content of an adult is 80 mg in total. Copper is absorbed from the diet in the duodenum, in chelated form with amino acids or small peptides. Absorbed copper enters the blood, links to the albumins and is transported to the liver, where it is incorporated into a protein - ceruloplasmin. This is secreted in the plasma, as the excess of copper is secreted in the bile. Ceruloplasmin cedes the copper to the cells after the fixation on the membranous receptors. The human body contains 200-400 mg/ l of ceruloplasmin, which includes 90% of the total plasmatic copper, the rest of plasmatic copper being bound to albumin, histidine and small peptides [**18**]. Ceruloplasmin has a peroxidase activity, oxidizing Fe2+ to Fe3+ and facilitating transferrin loading. The importance of this peroxidase activity of ceruloplasmin has been studied in patients with aceruloplasminemia, who had a mutation of the gene that codifies the protein. These patients have a low iron level, a high ferritin level and increasing of iron storage in brain and liver, causing diabetes, degenerative retinopathy and neurological disorder. The injection of ceruloplasmin increases the serum iron level [**19**]. Conversely, the iron oxidation catalyzed by the ceruloplasmin does not generate free radicals. Like an acute phase protein, ceruloplasmin can be a marker of inflammatory reaction and of pain. In investigated patients, the values presented in **Fig. 2** show an increase of ceruloplasmin level and of its activity during the dynamic of pain-inducing surgical treatment, values that remain in plateau for almost 7 days post-operatory, when the registered decrease is associated with a diminishing of the pain. It can be stated that this parameter biochemically reflects the verbally registered value in investigated patients.

**Fig. 3:**
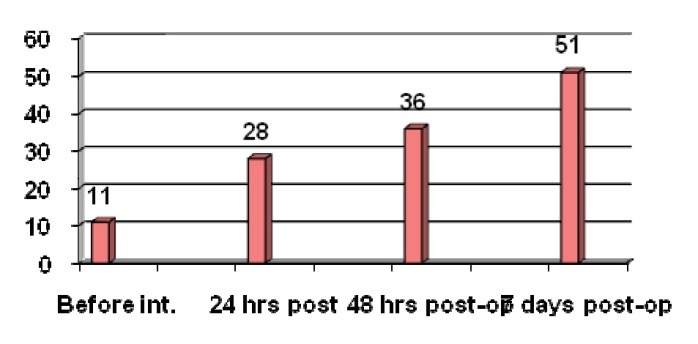
Dynamic of immune circulating complexes

Immune circulating complexes are formed as a consequence of binding the immunoglobulins (antibodies) to the antigens, having as a result a circulating antigen-antibody complex. Their role is only partially elucidated, being extremely useful in monitoring the effectiveness of treatment or of the disease evolution in cancer patients. For the investigated set of patients, we obtained the values of CIC presented in **Fig. 3**.

The registered values are in normal limits, the explanation being the pathogenicity and the manner of sedimentation on and in the membranes, which depends on the quantitative and qualitative aspects of them: the quantity of Atg-Atb complexes, the size of the complexes; the molecular configuration, antibody affinity for the antigen, electrical charge and solubility [**20**]. The big, insoluble complexes, formed in excess are fast removed from blood and phagocytized by the monocyte-macrophage system. On the other side, the intermediary size immune complexes formed in the excessive presence of some antigens remain in solution and they can be stored, delivering the values registered at 7 days post-operatory. Immune circulating complexes pathogenicity consists of their capacity to elicit a series of phenomena resulting in basal membranes alteration and cell proliferation [**21**]. The registered values are in concordance with the intensity of inflammatory processes and with the pain, thus, it can be affirmed that these determinations can also complete the clinical pain-associated picture in breast cancer female patients.

## Conclusions

The biochemical determinations performed for the measurement of lipid peroxides, ceruloplasmin and immune circulating complexes showed that these parameters can be useful in monitoring the pain intensity, which otherwise can be registered only in a subjective, verbally way. The chosen parameters are from different metabolic pathways, but all of them have a common point in the cascade of events associated with the inflammation and pain, and also with the events related to the malign transformation. Their monitoring in the dynamic of the anesthetic-surgical act becomes useful for an effective chasing and treatment of pain. Additionally, a new concept can be introduced, the perioperatory management of this type of patients.

The perioperatory management of patients with cancer disease can be a challenge, a science and an art. The multiple problems, directly related to the presence of the tumor but also to the antineoplastic treatments sequentially performed before and after the surgical procedure, induce the raising of morbidity and mortality risk during the perioperatory period. This period implies the passing of patients exposed to surgical treatment through three different physiological statuses: one preoperatory physiologic status, than the anesthetic intra-operatory and the post-operatory status, after that they should successfully return to the status anterior to the operation [**22**]. Days or even weeks are necessary for the recovery of physiological states from the perioperatory period. Any patient who presents a preoperatory pathology and must bear an elective surgical procedure, will pass these three physiopathological statuses under other doctors’ supervision: the internist or the oncologist in the perioperatory period, anesthesiologist and surgeon in the intra- and post-operatory period.

Oncological patients care exemplifies this concept of perioperatory medicine, combining knowledge and technical abilities of the anesthesiologist, radio/ chemotherapeutics oncologists and surgeons [**23**].

The perioperative medicine of the oncological patient needs the ability of integrating in a unitary vision the different problems of a patient generated by the presence of cancer disease, the performed treatments, but also the other associated non-malignant diseases, which add to the anesthetic risk.
